# Extracorporeal cardiopulmonary resuscitation following cardiac surgery: a scoping review

**DOI:** 10.1016/j.resplu.2025.101210

**Published:** 2025-12-30

**Authors:** Sho Takemoto, Tomonari M. Shimoda, Yuta Inoue, Hirofumi Kanazawa, Amir Sanatkar, Asishana Osho, Ryan Ruiyang Ling, Kollengode Ramanathan, Akira Shiose, Yohei Okada

**Affiliations:** a Center for Transplantation Sciences, Department of Surgery, Massachusetts General Hospital and Harvard Medical School, Boston, MA, USA; b Department of Cardiovascular Surgery, Kyushu University Graduate School of Medical Sciences, Fukuoka, Japan; c Department of Cardiovascular Surgery, University of Tsukuba, Ibaraki, Japan; d Department of Cardiovascular Surgery, Gifu University Graduate School of Medicine, Gifu, Japan; e Transplantation Research Center, Brigham and Women’s Hospital and Harvard Medical School, Boston, MA, USA; f Department of Anesthesiology, The University of Minnesota, Minneapolis, MN, USA; g Cardiac Surgery Division, Department of Surgery, Massachusetts General Hospital and Harvard Medical School, Boston, MA, USA; h Australian and New Zealand Intensive Care Research Centre, School of Public Health and Preventive Medicine, Monash University, Melbourne, Victoria, Australia; i Yong Loo Lin School of Medicine, National University of Singapore, National University Health System, Singapore, Singapore; j Department of Anaesthesia, Khoo Teck Puat Hospital, National Healthcare Group, Singapore, Singapore; k Cardiothoracic Intensive Care Unit, National University Heart Centre Singapore, National University Health System, Singapore, Singapore; l Department of Cardiovascular Surgery, Kyushu University Hospital, Fukuoka, Japan; m Department of Preventive Services, Kyoto University Graduate School of Medicine, Kyoto, Japan; n Prehospital and Emeregency Research Centre, Health Services Research and Population Health, Duke-NUS Medical School, National University of Singapore, Singapore, Singapore

**Keywords:** In-hospital cardiac arrest, Extracorporeal cardiopulmonary resuscitation, Extracorporeal membrane oxygenation, Cardiac surgery, Cardiac arrest following cardiac surgery, Special circumstances

## Abstract

**Background:**

In-hospital cardiac arrest after cardiac surgery demands specific approaches, such as rapid resternotomy, internal cardiac massage and sequential defibrillation. Extracorporeal cardiopulmonary resuscitation (ECPR) is a viable option; however, it is not standardized. This scoping review summarizes current evidence and identifies knowledge gaps regarding ECPR after cardiac surgery.

**Methods:**

We searched PubMed, Web of Science, Cochrane Library, and Ichushi-Web (a Japanese medical database) from July 29, 2024 through March 20, 2025. Studies reporting outcomes in patients received ECPR after cardiac surgery were included, without restrictions on study design or language.

**Results:**

Of 3963 unique articles, 49 studies were included. Among these, 3 adult and 17 pediatric studies reported ≥20 identifiable post–cardiac surgery ECPR cases (91 adult cases and 1464 pediatric cases). Across the included adult literature, only two ventricular assist device cases and no minimally invasive cardiac surgery cases were found. Adult post-cardiac surgery ECPR outcomes showed 33–35 % of overall survival and 23–29 % of survival with favorable neurological outcome. Pediatric outcomes were variable, with 10–70 % of overall survival and 10–40 % survival with favorable neurological outcome. Chest compression duration ranged from 31 to 36 min in adult post-cardiac surgery ECPR and from 27 to 60 min in pediatrics, where this metric was frequently available only from mixed surgical/non-surgical cohorts.

**Conclusion:**

Both adult and pediatric groups demonstrated variable but relatively high overall survival and survival with favorable neurological outcome following ECPR. Pediatric studies highlighted prolonged chest compressions. Further research is needed to explore the role of ECPR following minimally invasive and ventricular assist device surgery.

## Highlights

• First scoping map of post-cardiac-surgery extracorporeal cardiopulmonary resuscitation in adults and children.
• Overview of 49 studies capturing outcome ranges in post-cardiac-surgery ECPR.
• Major evidence gaps identified: non-sternotomy and ventricular assist device populations.
• Marked heterogeneity; protocols/timing need optimization.
• Actionable research roadmap to inform trials and guideline updates.

## Graphical abstract

**Figure:**
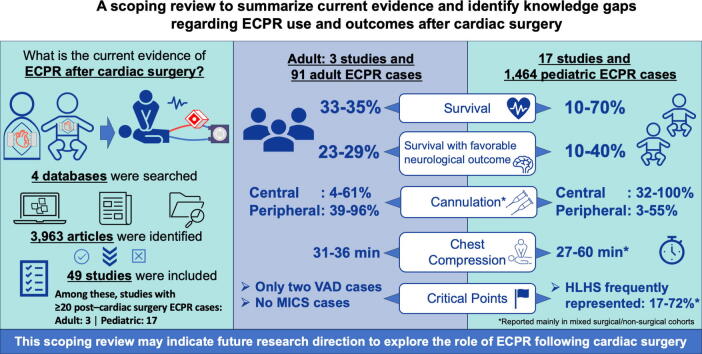

## Introduction

Cardiac arrest occurs in up to 8 % of patients following cardiac surgery.^1^ Particularly, once coronary flow is restored after the release of the aortic cross-clamp, the myocardium becomes sensitive to stimulation, increasing the risk of arrhythmias.^1^ Furthermore, cardiac arrest secondary to coronary complications such as myocardial ischemia or cardiac tamponade after cardiac surgery differs significantly from other types of in-hospital cardiac arrest (IHCA).^1^ Given the complexity and vulnerability of the post-cardiac surgery period, establishing clear and effective protocols to manage potential cardiac arrest following cardiac surgery is essential for ensuring patient safety.

Extracorporeal cardiopulmonary resuscitation (ECPR) remains a promising option worth considering for both adult and pediatric IHCA cases.^2^ However, its clinical role in IHCA following cardiac surgery remains controversial.^1^, ^3^ The European Resuscitation Council (ERC) advanced life support algorithm for cardiac arrest following cardiac surgery does not include a definitive recommendation for ECPR.^4^ In contrast, a joint expert consensus statement from the European Association of Cardio-Thoracic Surgery (EACTS), the Extracorporeal Life Support Organization (ELSO), the Society of Thoracic Surgeons (STS) and the American Association for Thoracic Surgery (AATS) have suggested standardized resuscitation strategies, including early resternotomy while continuing basic life support.^5^ This consensus statement recognizes ECPR as a potential option in specialized centers with skilled providers and the capacity for rapid ECMO deployment. However, immediate sternotomy may not be feasible in patients who have undergone non-sternotomy cardiac procedures, such as minimally invasive cardiac surgery (MICS). In such cases, ECPR could be still a viable option, despite the limited data.^4^

This scoping review aims to comprehensively map the existing literature on ECPR utilization, reported outcomes, and complications in both adult and pediatric patients who experience cardiac arrest following cardiac surgery, synthesizing the current evidence and identifying areas of uncertainty, and highlights key directions for future research to optimize ECPR application in these special circumstances.

## Materials and methods

### Protocol and registration

This scoping review follows the Preferred Reporting Items for Systematic Reviews and Meta-Analyses extension for Scoping Reviews (PRISMA-ScR) guidelines (Supplement A)^6^ and the methodological framework by Arksey and O’Malley, refined by Levac et al.^7^, ^8^ The protocol for this scoping review was not prospectively registered; methods are reported in detail in accordance with PRISMA-ScR.

### Eligibility criteria

The eligibility criteria are summarized using the Population–Concept–Context (PCC) framework in Table 1. We included peer-reviewed studies reporting outcomes in patients who underwent cardiac surgery and subsequently received ECPR. Secondary research articles, such as systematic reviews and meta-analyses, were excluded. No restrictions on study design or language were applied. Studies written in languages other than English or Japanese were considered if the content could be understood using translation applications such as DeepL Translator (DeepL, Cologne, Germany). The following types of manuscripts were also excluded: editorials, protocols, conference abstracts or posters, articles for which full texts were not available, and dissertations. For this review, “cardiac surgery” was defined as operative procedures performed for congenital or acquired cardiac disease (e.g., congenital repairs/palliation, valve surgery, coronary artery bypass grafting, open thoracic aortic surgery, ventricular assist device implantation, and heart transplantation), without restricting by cardiopulmonary bypass (CPB) use or surgical approach. Studies related to the following procedures were excluded: percutaneous procedures, including transcatheter valve replacement or repair, endovascular aortic repair, transapical aortic or mitral valve procedure, open abdominal aortic aneurysm repair.

**Table 1:** Summary of eligibility criteria for selecting studies.

	**Inclusion**	**Exclusion**
Population	• Patients who underwent cardiac surgery (e.g., coronary artery bypass grafting, valve surgery, heart transplant or ventricular assist device surgery, congenital heart surgery) • Patients who required ECPR post-cardiac surgery	• Patients who underwent non-cardiac surgery • Percutaneous procedures, including transcatheter valve replacement or repair, endovascular aortic repair, transapical aortic or mitral valve procedure, open abdominal aortic aneurysm repair • Cases of cardiogenic shock without cardiac arrest • Animal studies
Concept	• ECPR (veno-arterial ECMO initiated during ongoing chest compressions for refractory in-hospital cardiac arrest after cardiac surgery) • Studies that report any clinical outcomes (e.g., survival, neurological outcome, incidence of complications) • Case reports describing its clinical outcome	
Context	• Exploring outcomes of ECPR post-cardiac surgery	
Types of evidence	• Primary empirical research studies (e.g., randomized controlled trials, cohort studies, cross-sectional studies, and case reports) • Full-text articles	• Editorial articles • Protocols for planned studies • Abstracts or posters • Articles for which we cannot obtain the full text • Dissertations

ECMO: extracorporeal membrane oxygenation; ECPR: extracorporeal cardiopulmonary resuscitation.

### Handling of mixed cohorts

Because ECPR literature often includes mixed surgical and non-surgical cohorts (and/or broader ECMO cohorts), we prespecified that studies were eligible only when the post–cardiac surgery ECPR subgroup was explicitly identified in the abstract/full text and post–cardiac surgery subgroup outcomes were extractable. Studies that likely included post–cardiac surgery patients but did not explicitly report a post–cardiac surgery ECPR subgroup or allow extraction of subgroup outcomes were not included.

### Information sources and search strategy

Sources of evidence included PubMed, Web of Science, Cochrane Library, and Ichushi-Web (a Japanese medical database). The search strategy was developed by an experienced medical librarian team (Supplement B). The database search was conducted in July 2024 and updated in March 2025 without limitations on year to retain the full extent of available evidence. Deduplication was performed using EndNote 21 (Clarivate Analytics, Philadelphia, USA).

### Selection of sources of evidence, data extraction

We uploaded the articles to Rayyan and screened the titles and abstracts.^9^ Full texts of all potentially eligible studies were retrieved and reviewed by two authors against the eligibility criteria. Studies lacking online full text availability were excluded at this stage. Any disagreements in the selection process were resolved by discussion and consensus of the authors. We then extracted the data using standardized data sheet. The data collection is summarized in Supplement C. To structure the presentation of findings, data related to the ECPR process were described according to a conceptual timeline framework comprising four key phases: Phase 1: Decision-Making and Pre-ECMO Resuscitation; Phase 2: ECMO Deployment; Phase 3: On-ECMO Management and Stabilization; and Phase 4: Weaning, Decannulation, and Post-ECMO Care. Screening of articles and data collection were done by ST, TS, YI and HK, and full-text review was done by ST and TS, all independently and in duplicate.

### Data items: Definition of ECPR and favorable neurological outcomes

In this review, ECPR is defined as veno-arterial ECMO initiated during ongoing chest compressions for refractory IHCA after cardiac surgery; ECMO instituted without active cardiopulmonary resuscitation (CPR) for postcardiotomy low-output states or shock, and ECMO initiated intraoperatively during the index surgery, were considered postoperative/intraoperative ECMO and were not counted as ECPR in our analyses. We classified survivors as having a favorable neurological outcome when authors reported survival or discharge without major neurological complications; this included cases with minor imaging-only abnormalities in the absence of clinically significant deficits.

### Critical appraisal of individual sources of evidence

No formal critical evaluation of risk of bias was undertaken based on the methodology of scoping reviews.^7^, ^10^

### Synthesis of results

We conducted a descriptive synthesis using counts, proportions, and study-level ranges, without pooling estimates or calculating summary measures across studies. For the main summary tables, we prioritized studies with ≥20 identifiable post–cardiac surgery ECPR cases and extractable post-cardiac surgery subgroup outcomes; studies with an identifiable but smaller post-cardiac surgery subgroup (<20) and case reports/series were summarized in the supplemental tables.

## Results

### Study selection

**Fig. 1:**
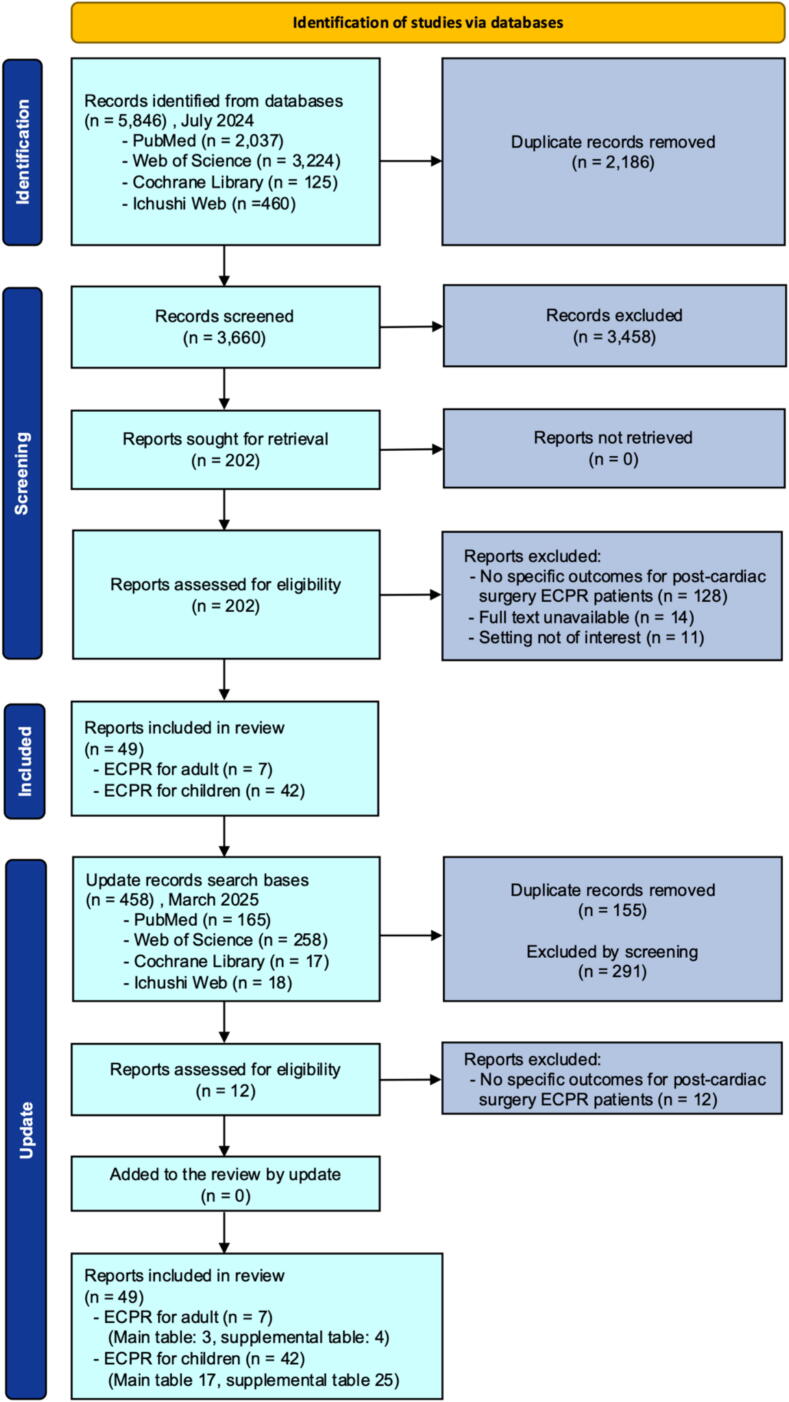
**PRISMA flowchart**. ECPR: extracorporeal cardiopulmonary resuscitation.

After removing duplicates, 3963 articles were screened for title and abstract (Fig. 1). Finally, 49 papers (seven articles on adult^10^, ^11^, ^12^, ^13^, ^14^, ^15^, ^16^ and 42 articles on pediatric^17^, ^18^, ^19^, ^20^, ^21^, ^22^, ^23^, ^24^, ^25^, ^26^, ^27^, ^28^, ^29^, ^30^, ^31^, ^32^, ^33^, ^34^, ^35^, ^36^, ^37^, ^38^, ^39^, ^40^, ^41^, ^42^, ^43^, ^44^, ^45^, ^46^, ^47^, ^48^, ^49^, ^50^, ^51^, ^52^, ^53^, ^54^, ^55^, ^56^, ^57^, ^58^) were included. Among these, 3 adult^10^, ^11^, ^12^ and 17 pediatric^17^, ^18^, ^19^, ^20^, ^21^, ^22^, ^23^, ^24^, ^25^, ^26^, ^27^, ^28^, ^29^, ^30^, ^31^, ^32^, ^33^ studies reported ≥20 identifiable post–cardiac surgery ECPR cases (91 adult cases and 1464 pediatric cases).

### Phase 1: Decision-making and pre-ECMO resuscitation

#### Patient population and procedural characteristics

##### Adult studies

Table 2 summarizes patient and procedural characteristics. Among the three adult studies with ≥20 identifiable post–cardiac surgery ECPR cases, the range of mean age was 57–62 years.^10^, ^11^, ^12^ Surgical procedures included a total of 42 (46 %) isolated coronary artery bypass grafting cases, 26 (29 %) valve surgeries, and two cases involving left ventricular assist device (LVAD).^10^, ^11^, ^12^, ^13^, ^14^, ^15^, ^16^ No reports of ECPR following MICS were identified. Studies with identifiable post-cardiac surgery ECPR subgroup (*n* < 20)^13^, ^14^ and case reports^15^, ^16^ were summarized in Tables S1 and S2.

**Table 2:** Summary of included studies: Adult studies with identifiable post-cardiac surgery ECPR subgroup (*n* ≥ 20).

**Author/Year**	**Age, years**	**Isolated CABG**	**Valve surgery**	**VAD surgery**	**MICS**	**Cause of cardiac arrest**	**Post-cardiac surgery ECPR**	**Survival in post-cardiac surgery ECPR patients**	Survival with favorable neurological outcome in post-cardiac surgery ECPR patients, *n* (%)*	**Other ECMO-related complications**	**Duration of chest compression, min**	**Cannulation site**
Levy/2022	62 ± 11	15 (34)	12 (27) (isolated valve) 7 (16) (CABG + valve)	1 (2)	0 (0)	PEA (unclear cause) 22 (50) Ventricular failure 8 (18) Tamponade 2 (4.5)	**44 (100)**	**15/44 (34)**	**11 (25)**	Sternal infection 2 (5) Distal ischemia 6 (14) Other (cardiac, MOF, sepsis) 20 (46)	36 ± 23	Central 26 (59) Peripheral 18 (41)
Mazzeffi/2016	57 ± 15	7 (30)	5 (22)	0 (0)	0 (0)	Tamponade 4 (17) Right ventricular failure 3 (13) Coronary artery obstruction 3 (13) Respiratory arrest 2 (8.7) Biventricular failure 1 (4.4) Severe acidosis 1 (4.4) Unclear 9 (39)	**23 (100)**	**8 /23 (35)**	**6 (23)**	Dialysis 11 (48)	31 (15–52)	Central 14 (61) Peripheral 9 (39)
Zhao/2015	59 ± 12	20 (83)	2 (8)	0 (0)	0 (0)	NA	**24 (100)**	**8 /24 (33)**	**7 (29)**	Tamponade 4 (17) Infection 11 (46) GI bleeding 5 (21) Dialysis 7 (29) Distal ischemia 2 (8) CPR (29)MOF 12 (50)	36 (NA)	Central 1 (4) Peripheral 23 (96)

Values are *n*, *n* (%), median (25–75 %tile), or mean (SD). Values are presented as reported in the primary studies and were not transformed. NA (not applicable) indicates the variable was not reported at the study/stratum level. All studies listed were retrospective/observational.

CABG: coronary artery bypass graft; CPR: cardiopulmonary resuscitation; ECMO: extracorporeal membrane oxygenation; ECPR: extracorporeal cardiopulmonary resuscitation; GI: gastrointestinal; MICS: minimally invasive cardiac surgery; MOF: multiple organ failure; NA: not applicable; PEA: pulseless electrical activity; VAD; ventricular assist device.

Bolded values indicate numbers and outcomes specific to the post–cardiac surgery (postcardiotomy) ECPR subgroup.

* Includes patients reported as “discharged from hospital” or “discharged to home” where specific details of neurological outcome were not provided.

##### Pediatric studies

Pediatric studies with post-cardiac ECPR (*n* ≥ 20) are summarized in Table 3.^17^, ^18^, ^19^, ^20^, ^21^, ^22^, ^23^, ^24^, ^25^, ^26^, ^27^, ^28^, ^29^, ^30^, ^31^, ^32^, ^33^ Range of median or mean age was 0 day–5.2 years. Studies with identifiable post-cardiac surgery ECPR subgroup (*n* < 20) and case reports/series were summarized in Tables S3 and S4. Among 33 observational studies, nine studies reported that hypoplastic left heart syndrome (HLHS) accounted for the largest proportion of cases, with rates ranging 17–72 %.^20^, ^30^, ^33^, ^34^, ^36^, ^39^, ^41^, ^42^, ^59^

**Table 3:** Summary of included studies: Pediatric studies with identifiable post-cardiac surgery ECPR subgroup (*n* ≥ 20).

**Authors**	Total cohort size*	**Population**	**Age**	**Body weight, kg**	**Single ventricular physiology**	**HLHS or Norwood-type operation**	**Post-cardiac surgery ECPR**	**Survival in post-cardiac surgery ECPR patients**	Survival with favorable neurological outcome in post-cardiac surgery ECPR**	**Duration of chest compression, min**	**Cannulation site**
Alsoufi/2009	180	Post-cardiac surgery ECMO	109 d (14–465)	4.3 (3–9)	69 (34)	NA	**48**	**22/48 (46)**	**NA**	NA	Central 168 (93) Neck 12 (7)
Alsoufi/2014	39	Post-cardiac surgery ECPR	44 (20–160)d	4 (3–5)	13 (33)	NA	**39**	**16/39 (41)**	**NA**	34 (8–125)	NA
Anton-Martin/2020	73	ECPR including non-surgical patients	175 (13–730) d	5 (3.5–13)	30 (41)	NA	**35**	**14/35 (40)**	**NA**	56 (45–81)	Central 45 % Peripheral 55 %
Basgoze /2022	109	Post-cardiac surgery ECMO	10 ± 21 mo	6.4 ± 6.8	40 (37)	18 (17)	**31**	**3/31 (10)**	**3 (10)**	NA	NA
Beshish/2018	80	ECPR including non-surgical patients	1.3 mo (0–11)	NA	33 (41)	NA	**61**	**30/61 (49)**	**26 (32)**	57 ± 24	Central 44 (55) Percutaneous 36 (45)
Brown/2023	124	ECPR including non-surgical patients	0.9 (0.2–5) y	NA	28 (23)	NA	**52**	**30/52 (57)**	**NA**	47 (36–60)	Central 38 (32) Neck 58 (50)
Chan/2008	492	ECPR including non-surgical patients	80 (12–452) d	4.3 (3.2–9.5)	196 (40)	NA	**279**	**115/279 (41)**	**NA**	NA	Central 216 (44)
Erek/2017	25	Post-cardiac surgery ECMO	3 mo (2 d–4.5 y)	NA	NA	2 (8)	**25**	**5/25 (20)**	**4 (16)**	<20 min 2; 20–40 min 11; >40 min 12	Central 25 (100)
Huang/2012	54	ECPR including non-surgical patients	5.2 ± 5.9 y	NA	NA	1 (1)	**22**	**7/22 (31)**	**21 (39)**	45 ± 35	Number not described. Post-cardiac surgery: central cannulation; others: peripheral.
Jin/2020	85	Post-cardiac surgery ECMO	12.7 (6–43) m	8.5 (6–13)	NA	NA	**21**	**5/21 (23)**	**NA**	NA	Central 82 (96) Peripheral 3 (4)
Kane/2010	172	ECPR including non-surgical patients	5.7 mo (0.4–43)	6.0 (3–14)	70 (41)	NA	**103**	**56/103 (54)**	**69 (40)**	33 (23–44)	Arterial: Aorta 92 (53) Right carotid 46 (26) ; other 34 (19). Venous: Right atrium 91 (52); Right internal jugular 47 (27); other 34 (19). Thoracic cannulation 96 (55).
Kobayashi/2024	1223	C-CPR or ECPR (including non-surgical patients)	Median: NA <1 mo: 39 % 1 mo–<1 y: 35 % 1–8 y: 17 % 9–17 y: 9 %	4.5 (3.1–8.4)	NA	NA	**482**	**228/482 (47)**	**NA**	42 (28–58)	NA
Melvan/2020	184	ECPR including non-surgical patients	54 (11–272) d	4 (3.0–8.5)	71 (38)	NA	**124**	**53/124 (43)**	**49 (40)**	27 (17–39)	Central 107 (58) Peripheral 77 (42)
Shah/2005	84	Post-cardiac surgery ECMO	128 d (NA)	4.5 (2–18)	47 (56)	29 (35)	**27**	**9/27 (33)**	**NA**	NA	Central 80 (95) Neck 4 (5)
Torres-Andres/2018	58	ECPR including non-surgical patients	3.5 mo (1–53)	6 (3–20)	NA	NA	**24**	**17/24 (70)**	**NA**	31 (20 – 49)	Central 19 (32)Peripheral (neck) 32 (55) Neck + groin 5 (8) Central + peripheral 2 (3)
Walter/2011	42	ECPR including non-surgical patients	0.7 (NA) y	7.0 (NA)	3 (7)	2 (4.7)	**27**	**9/27 (33)**	**NA**	45 ± 4	Central 37 (86) Peripheral 5 (14)
Wolf/2012	90	ECPR including non-surgical patients	2.0 (NA) y	NA	41 (46)	18 (20)	**64**	**30/64 (47)**	**NA**	Survivors: 45 ± 17 Nonsurvivors: 42 ± 14	Central 60 (67) Peripheral 30 (33) (neck 23 [26], femoral 7 [8])

Values are *n*, *n* (%), median (25–75 %tile), or mean (SD). Values are presented as reported in the primary studies and were not transformed. NA (not applicable) indicates the variable was not reported at the study/stratum level.

C-CPR: conventional cardiopulmonary resuscitation; ECMO: extracorporeal membrane oxygenation; ECPR: extracorporeal cardiopulmonary resuscitation; HLHS: hypoplastic left heart syndrome; NA: not applicable.

Bolded values indicate outcomes specific to the post–cardiac surgery (postcardiotomy) ECPR subgroup (i.e., calculated using the post–cardiac surgery ECPR denominator when extractable). All other values reflect the overall study cohort unless otherwise specified.

* Include the total number of patients in the source study including non-ECPR patients.

** Includes patients reported as “discharged from hospital” or “discharged to home” where specific details of neurological outcome were not provided. More details and definition of favorable neurological outcome were summarized in Table S5.

##### Duration of chest compression

**Fig. 2:**
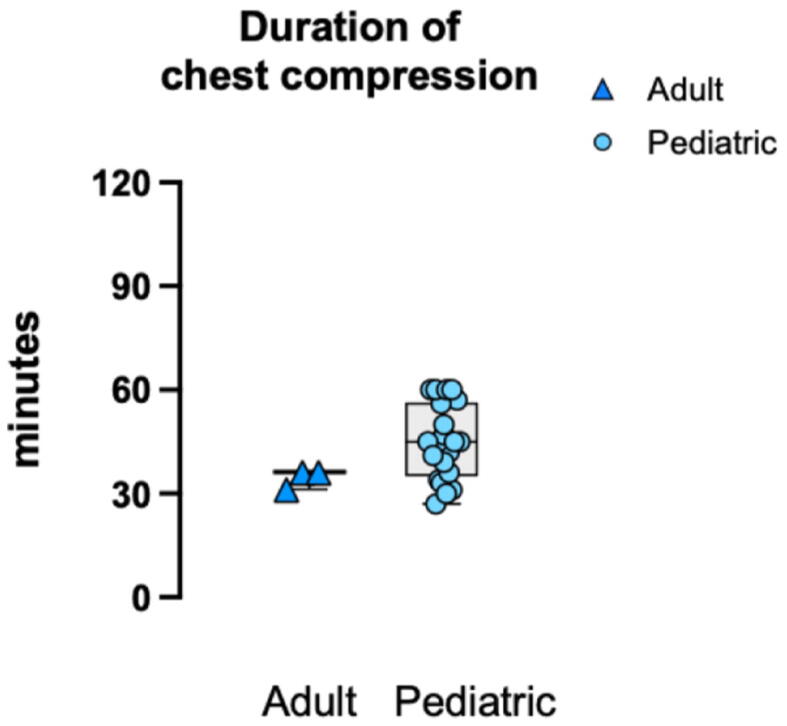
**Duration of chest compression**. Reported durations are shown as provided in each study (median or mean). Because post-cardiac surgery ECPR–specific chest compression duration was seldom available, values include mixed cohorts (surgical and non-surgical populations) when subgroup-specific data were not reported. ECPR: extracorporeal cardiopulmonary resuscitation.

Median or mean chest compression duration ranged 31–36 min for adult (Fig. 2).^10^, ^11^, ^12^ For pediatric patients, median or mean chest compression duration ranged 27–60 min (Fig. 2).^18^, ^19^, ^21^, ^22^, ^25^, ^37^, ^27^, ^28^, ^29^, ^32^, ^33^, ^34^, ^39^, ^40^, ^41^, ^42^, ^43^, ^45^, ^46^, ^47^, ^48^.

### Phase 2: ECMO deployment

#### Cannulation strategies

**Fig. 3:**
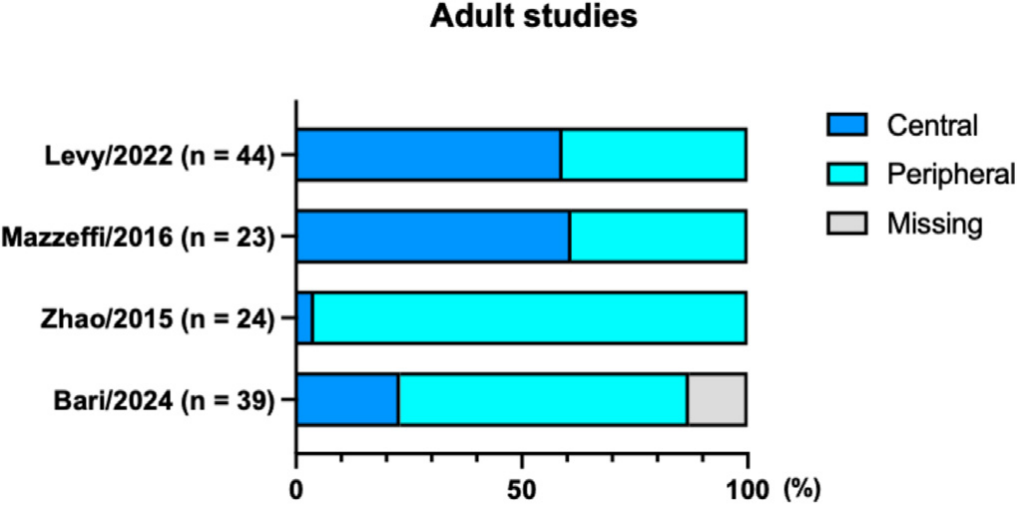
**Cannulation site distribution in adultstudies.** (*n* = number of patients underwent extracorporeal membrane oxygenation or resuscitation in each study).

**Fig. 4:**
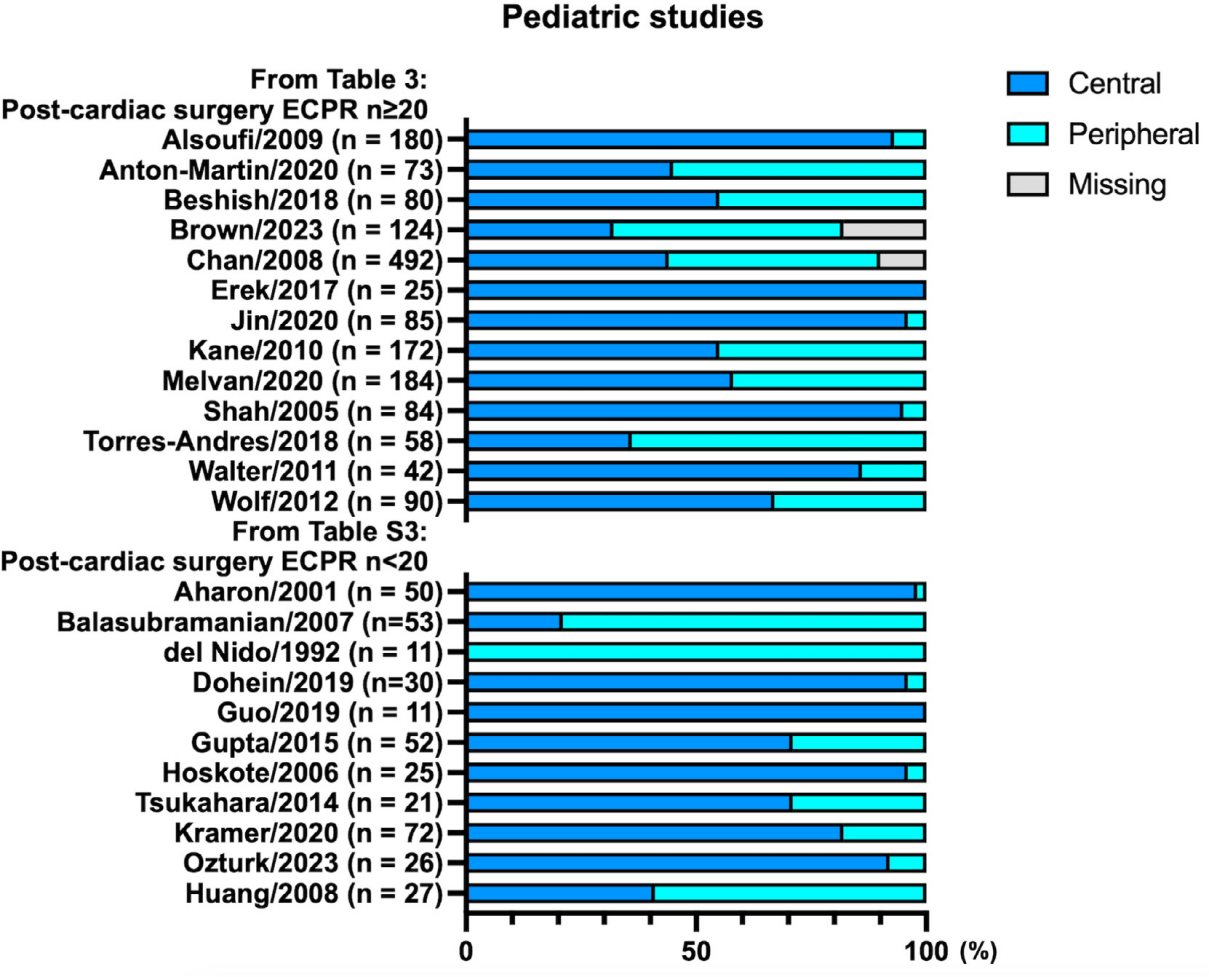
**Cannulation site distribution in pediatricstudies.** (*n* = number of patients underwent extracorporeal membrane oxygenation or resuscitation in each study).

In the four adult observational studies, central cannulation was used in 4–61 %, and peripheral in 39–96 % of cases.^10^, ^11^, ^12^, ^13^ In pediatric studies, central cannulation was used in 32–100 %, while peripheral (mostly neck) in 3–55 % of cases.^18^, ^19^, ^26^, ^27^, ^37^, ^21^, ^22^, ^23^, ^24^, ^29^, ^30^, ^31^, ^32^, ^33^, ^34^, ^35^, ^39^, ^40^, ^41^, ^42^, ^45^, ^46^, ^47^, ^48^ Cannulation site distribution is summarized in Fig. 3, Fig. 4.

### Phase 3: On-ECMO management and stabilization

#### Complications during ECPR and subsequent ECMO support

In pediatric observational studies, bleeding complications (re-exploration, gastrointestinal and cannulation site bleeding) were noted in 11 studies, with incidences ranging 1–100 %.^17^, ^20^, ^23^, ^29^, ^30^, ^32^, ^34^, ^37^, ^42^, ^43^, ^59^ Other ECMO-related complications included infection (sepsis, pulmonary infection, mediastinitis) (1–51 %),^17^, ^20^, ^23^, ^32^, ^34^, ^37^, ^40^, ^42^, ^43^ dialysis (8–68 %),^17^, ^20^, ^23^, ^29^, ^32^, ^34^, ^42^, ^43^, ^59^ necrotizing enterocolitis (6–36 %),^43^, ^59^ ECMO circuit or mechanical related problem (consumption coagulopathy, circuit thrombus, oxygenator exchange, and cannula dislodgement) (12–22 %),^17^, ^29^, ^30^, ^32^, ^40^, ^59^ and MOF (29–36 %)^42^, ^43^ (Tables S3–S5).

### Phase 4: Weaning, decannulation, and post-ECMO care

#### Overall survival and survival with favorable neurological outcome

##### Adult outcomes

**Fig. 5:**
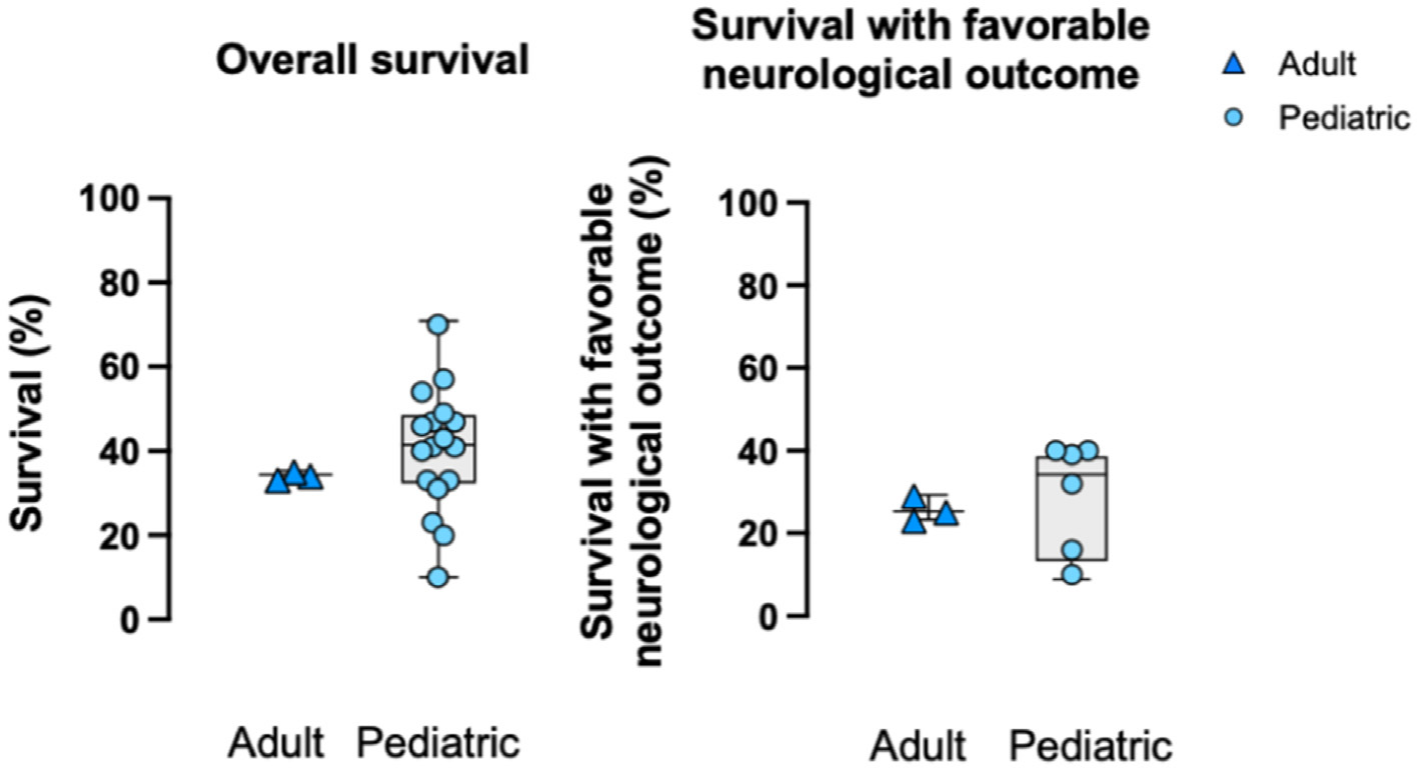
**Reported overall survival and survival with favorable neurological outcome in post-cardiac surgeryECPR.** Shown are study-level ranges restricted to studies with ≥20 identifiable post–cardiac surgery ECPR cases and extractable post–cardiac surgery subgroup outcomes. Favorable neurological outcome is reported as defined by each study. ECPR: extracorporeal cardiopulmonary resuscitation.

Among the three adult observational studies focused on post-cardiac surgery ECPR, overall survival ranged 33–35 % and survival with favorable neurological outcome ranged 23–29 % (Table 2, Fig. 5).^10^, ^11^, ^12^.

##### Pediatric outcomes

In the extracted pediatric post-cardiac surgery ECPR population, overall survival ranged 10–70 %^17^, ^18^, ^19^, ^20^, ^21^, ^22^, ^23^, ^24^, ^25^, ^26^, ^27^, ^28^, ^29^, ^30^, ^31^, ^32^, ^33^ and survival with favorable neurological outcome ranged 10–40 %^20^, ^21^, ^24^, ^25^, ^27^, ^29^ (Table 3, Fig. 5).

## Discussion

This scoping review summarizes current evidence on ECPR following cardiac surgery in adult and pediatric populations. No randomized trials were identified, and all included studies were retrospective. Evidence remains limited in adults compared with pediatrics, and reported overall survival and neurologically favorable survival varied widely across studies but were not uniformly poor.

In adults, the available evidence is insufficient to determine whether ECPR improves outcomes compared with conventional CPR after cardiac surgery. Reported survival for ECPR in broader IHCA populations including non-surgical patients is approximately 20–40 %,^60^, ^61^ whereas cardiac surgery-associated arrests have high mortality of 30–80 % with conventional resuscitation.^62^, ^63^, ^64^ These comparisons should be interpreted cautiously because case mix differs across settings, including differences in initial rhythm distribution among survivors.^60^ Direct comparative studies of ECPR versus conventional resuscitation specifically after cardiac surgery remain lacking.

In post-cardiac surgery arrest, surgically correctable causes are relatively common, which provides the rationale for cardiac surgery–specific resuscitation algorithms.^5^ However, postoperative-specific arrest etiologies were not reported consistently across included studies, limiting subgroup inference and highlighting the need for standardized reporting.

A key role of post-cardiac surgery ECPR may be to bridge to surgical intervention, maintaining cerebral, coronary, and end-organ perfusion. This aligns with expert consensus guidelines that prioritize prompt resternotomy within 5 min when initial defibrillation or pacing attempts fail.^1^, ^3^, ^4^, ^5^ In practice, achieving truly rapid ECMO deployment remains challenging, as adult studies reported chest compression durations of 31–36 min before ECMO initiation.^10^, ^11^, ^12^.

Establishing CPB following rapid resternotomy rather than initiating ECMO can be an alternative option, which further complicates the decision-making process, but no comparative studies were identified in our search. Institutional volume and experience are crucial for optimizing outcomes, suggesting the importance of developing dedicated, high-volume ECPR programs and team.^65^ Furthermore, the timing of intervention appears critical; the Post-cardiotomy Extracorporeal Life Support (PELS) group reported lower in-hospital mortality when ECMO was initiated intraoperatively compared to initiation in the intensive care unit, where indications included cardiac arrest. The PELS data highlight the importance of preventing cardiac arrest by intervening earlier.^66^

Our review also highlights a critical lack of procedure-specific evidence in increasingly common adult contexts, including MICS and VAD implantation. Although ECPR could be particularly relevant when standard approaches are difficult, existing literature is sparse, and resuscitation strategies in VAD patients remain insufficiently standardized. While VAD manufacturers and many hospitals advise against chest compressions in VAD patients,^67^ a few observational studies reported that device dislodgement was not consistently observed when chest compressions were performed.^67^, ^68^, ^69^ This significant gap emphasizes the need for dedicated research to evaluate the applicability, safety, and outcomes of ECPR in these specific population.

In contrast to adults, resternotomy in children, particularly infants and younger children, is less technically challenging. However, peripheral cannulation, a common approach in adult ECPR, is much more challenging in smaller pediatric patients due to the small size of femoral and carotid vessels. Therefore, cannulation often requires cutdown for direct visualization of those vessels. This inherently more invasive and time-consuming procedure may contribute to the higher proportion of central cannulation via resternotomy, and the longer chest compression durations reported in pediatric ECPR studies.

Despite the prolonged resuscitation times, pediatric studies did not consistently exhibit worse outcomes than adult series. Registry data suggest that children with cardiac arrest after cardiac surgery may tolerate for prolonged resuscitation better than other pediatric IHCA etiologies.^70^ Intact survival could be achieved in 17 % of the subset of chest compressions exceeding 35 min, suggesting a potentially different physiological response to prolonged ischemia and reperfusion in this specific clinical context.^70^

Taken together, our review identifies pediatric-specific knowledge gaps that limit optimization of post–cardiac surgery ECPR. Postoperative subgroup reporting is inconsistent, with frequent reliance on mixed surgical/non-surgical or broader ECMO cohorts that precludes reliable postoperative risk stratification. Neurological outcome reporting is also non-standardized, with variable definitions and assessment time points, despite its importance for long-term survivorship. Moreover, patient selection and modifiable process metrics that could guide escalation to ECPR are seldom reported in a uniform manner, limiting generalizability.

**Fig. 6:**
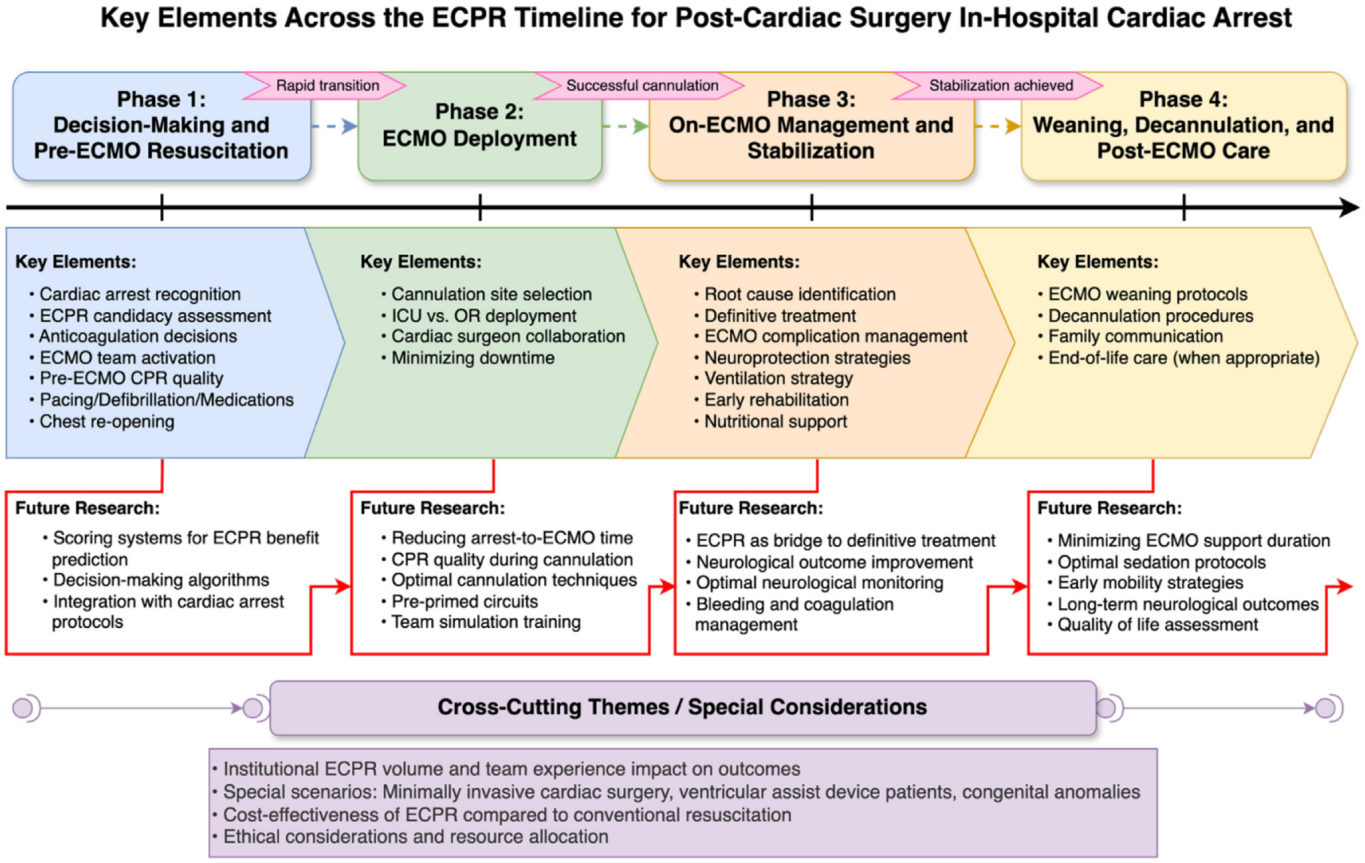
**Key elements across the ECPR timeline and areas of future research**. CPR: cardiopulmonary resuscitation; ECMO: extracorporeal corporeal membrane oxygenation; ECPR: extracorporeal cardiopulmonary resuscitation.

Within these constraints, available evidence reinforces the need to refine resuscitation processes. Lauridsen et al. reported each 5-second increment in longest chest compression pause duration during pediatric IHCA was associated with lower chance of survival with favorable neurological outcome, survival to hospital discharge, and return of spontaneous circulation.^71^ Kobayashi et al. reported that ECPR was associated with a higher rate of poor neurological outcomes (19 % in ECPR vs. 9 % in conventional CPR).^28^ These findings highlight the ongoing efforts to optimize every aspect of the ECPR process. Our proposed framework outlines crucial considerations and identifies specific areas for future research at each phase of ECPR (Fig. 6). This structured approach aims to guide efforts in improving ECPR strategies, enhancing team performance, and ultimately, refining patient selection to improve outcomes for both adult and pediatric patients undergoing ECPR after cardiac surgery.

### Limitation

This scoping review has several limitations. As a scoping review, our methodology was designed to broadly map the existing evidence rather than conduct an in-depth critical appraisal of individual studies. Therefore, we did not formally assess the risk of bias within included studies, and the quality of evidence varies considerably across the reviewed literature. Additionally, due to the inherent susceptibility to publication bias, studies and cases with negative findings may be underrepresented. Furthermore, this study did not incorporate quantitative analyses such as integrating the estimate of prevalence. Postoperative-specific synthesis was further limited because many pediatric reports presented mixed cohorts and did not consistently provide extractable post–cardiac surgery ECPR subgroup data. To avoid assuming subgroup composition, we did not include studies in which postoperative subgroup outcomes could not be explicitly identified, which may have resulted in omission of some relevant mixed-cohort evidence.

## Conclusion

This scoping review provides a comprehensive mapping of the current literature on ECPR following cardiac surgery in both adult and pediatric patients. Our synthesis confirms that the evidence base is limited and heterogeneous, characterized by variable survival and neurological outcomes in both populations. This review therefore highlights critical knowledge gaps regarding optimal patient selection criteria, standardized ECPR protocols. The lack of evidence is particularly pronounced for specific subgroups, such as patients undergoing MICS and those with VADs. Future research is essential to investigate the applicability, safety, and outcomes of ECPR in these populations. Also, more robust data are needed to clarify the role of ECPR and optimize its application in congenital heart surgery.

## Declaration of Generative AI and AI-assisted technologies in the writing process

During the preparation of this work the author(s) used Gemini 2.5 (Google, Inc. CA, USA) to enhance the manuscript's readability and clarity. After using this tool/service, the author(s) reviewed and edited the content as needed and take(s) full responsibility for the content of the publication.

## CRediT authorship contribution statement

**Sho Takemoto:** Writing – original draft, Visualization, Investigation, Formal analysis, Data curation, Conceptualization. **Tomonari M. Shimoda:** Writing – review & editing, Investigation, Data curation. **Yuta Inoue:** Writing – review & editing, Investigation, Data curation. **Hirofumi Kanazawa:** Writing – review & editing, Investigation, Data curation. **Amir Sanatkar:** Writing – review & editing, Investigation, Data curation. **Asishana Osho:** Writing – review & editing, Supervision, Project administration, Methodology, Conceptualization. **Ryan Ruiyang Ling:** Writing – review & editing, Supervision, Project administration, Methodology, Conceptualization. **Kollengode Ramanathan:** Writing – review & editing, Supervision, Project administration, Methodology, Conceptualization. **Akira Shiose:** Writing – review & editing, Supervision, Project administration, Methodology, Conceptualization. **Yohei Okada:** Writing – review & editing, Validation, Supervision, Project administration, Methodology, Conceptualization.

## Funding

No funding was obtained for this study.

## Declaration of competing interest

The authors declare the following financial interests/personal relationships which may be considered as potential competing interests: YO received the research fund from the ZOLL foundation, the KPFA research fellowship, JSPS-Overseas fellowship. These funds have no role to conduct this review. RRL acknowledges research funding support outside of this work from the Clinician Scientist Development Unit, Yong Loo Lin School of Medicine. He is an editorial board member of *Critical Care* and is a fellow of the ELSO Scientific Oversight Committee. KR is the chair of the ELSO Publications Committee, serves on the Steering Committee of ELSO, and is the immediate past co-chair of the ELSO Scientific Oversight Committee.

## Data Availability

The data underlying this article will be shared on reasonable request to the corresponding author.
