# Supplementary material for: Extracorporeal cardiopulmonary resuscitation following cardiac surgery: a scoping review
Source: Resusc Plus. 2025 Dec 30;27:101210. doi: 10.1016/j.resplu.2025.101210 (PMC12825076; doi:10.1016/j.resplu.2025.101210)
Supplement: Supplementary Data 1 [file mmc1.docx]

Supplement A: Preferred Reporting Items for Systematic reviews and Meta-Analyses extension for Scoping Reviews (PRISMA-ScR) Checklist

| **SECTION** | **ITEM** | **PRISMA-ScR CHECKLIST ITEM** | **REPORTED ON PAGE #** |
| --- | --- | --- | --- |
| **TITLE** | | | |
| Title | 1 | Identify the report as a scoping review. | Page 1 |
| **ABSTRACT** | | | |
| Structured summary | 2 | Provide a structured summary that includes (as applicable): background, objectives, eligibility criteria, sources of evidence, charting methods, results, and conclusions that relate to the review questions and objectives. | Page 2 |
| **INTRODUCTION** | | | |
| Rationale | 3 | Describe the rationale for the review in the context of what is already known. Explain why the review questions/objectives lend themselves to a scoping review approach. | Page 4 |
| Objectives | 4 | Provide an explicit statement of the questions and objectives being addressed with reference to their key elements (e.g., population or participants, concepts, and context) or other relevant key elements used to conceptualize the review questions and/or objectives. | Page 4 |
| **METHODS** | | | |
| Protocol and registration | 5 | Indicate whether a review protocol exists; state if and where it can be accessed (e.g., a Web address); and if available, provide registration information, including the registration number. | Page 5 Registration: NA |
| Eligibility criteria | 6 | Specify characteristics of the sources of evidence used as eligibility criteria (e.g., years considered, language, and publication status), and provide a rationale. | Page 5 and Table 1 |
| Information sources* | 7 | Describe all information sources in the search (e.g., databases with dates of coverage and contact with authors to identify additional sources), as well as the date the most recent search was executed. | Page 6 |
| Search | 8 | Present the full electronic search strategy for at least 1 database, including any limits used, such that it could be repeated. | Page 8 and supplement B |
| Selection of sources of evidence† | 9 | State the process for selecting sources of evidence (i.e., screening and eligibility) included in the scoping review. | Page 8 and Figure 1 |
| Data charting process‡ | 10 | Describe the methods of charting data from the included sources of evidence (e.g., calibrated forms or forms that have been tested by the team before their use, and whether data charting was done independently or in duplicate) and any processes for obtaining and confirming data from investigators. | Page 8 |
| Data items | 11 | List and define all variables for which data were sought and any assumptions and simplifications made. | Page 8 and supplement C |
| Critical appraisal of individual sources of evidence§ | 12 | If done, provide a rationale for conducting a critical appraisal of included sources of evidence; describe the methods used and how this information was used in any data synthesis (if appropriate). | NA |
| Synthesis of results | 13 | Describe the methods of handling and summarizing the data that were charted. | Page 7 |
| **RESULTS** | | | |
| Selection of sources of evidence | 14 | Give numbers of sources of evidence screened, assessed for eligibility, and included in the review, with reasons for exclusions at each stage, ideally using a flow diagram. | Page 8 and Figure 1 |
| Characteristics of sources of evidence | 15 | For each source of evidence, present characteristics for which data were charted and provide the citations. | Page 8 |
| Critical appraisal within sources of evidence | 16 | If done, present data on critical appraisal of included sources of evidence (see item 12). | NA |
| Results of individual sources of evidence | 17 | For each included source of evidence, present the relevant data that were charted that relate to the review questions and objectives. | Pages 8-10 and table 2-3 |
| Synthesis of results | 18 | Summarize and/or present the charting results as they relate to the review questions and objectives. | Pages 8-10 and Figure 2-5 |
| **DISCUSSION** | | | |
| Summary of evidence | 19 | Summarize the main results (including an overview of concepts, themes, and types of evidence available), link to the review questions and objectives, and consider the relevance to key groups. | Pages 11-15 |
| Limitations | 20 | Discuss the limitations of the scoping review process. | Page 13-14 |
| Conclusions | 21 | Provide a general interpretation of the results with respect to the review questions and objectives, as well as potential implications and/or next steps. | Page 14 |
| **FUNDING** | | | |
| Funding | 22 | Describe sources of funding for the included sources of evidence, as well as sources of funding for the scoping review. Describe the role of the funders of the scoping review. | Page 15 |

Supplement B: Search strategies for the scoping review

| ***PubMed/MEDLINE*** | | |
| --- | --- | --- |
|  | Query | Records on July 29^th^, 2024 |
| #1 | "cardiac surgical procedures"[MeSH Terms] | 251,497 |
| #2 | "cardiac surg*"[Title/Abstract] | 59,013 |
| #3 | "heart surg*"[Title/Abstract] | 22,533 |
| #4 | "cardiac operati*"[Title/Abstract] | 4,637 |
| #5 | "heart operati*"[Title/Abstract] | 1,371 |
| #6 | "Cardiopulmonary Resuscitation"[Mesh] | 23,341 |
| #7 | "Cardiopulmonary Resuscitation*"[Title/Abstract] | 20,683 |
| #8 | "CPR"[Title/Abstract] | 16,903 |
| #9 | "Cardiopulmonary Bypass"[Mesh] | 25,474 |
| #10 | "Cardiopulmonary Bypass"[Title/Abstract] | 36,767 |
| #11 | #1 or #2 or #3 or #4 or #5 or #6 or #7 or #8 or #9 or #10 | 338,655 |
| #12 | "Heart Arrest"[Mesh] | 58,244 |
| #13 | "Heart Arrest*"[Title/Abstract] | 2,615 |
| #14 | "cardiac arrest*"[Title/Abstract] | 47,601 |
| #15 | "asystole*"[Title/Abstract] | 4,365 |
| #16 | "Cardiopulmonary Arrest*"[Title/Abstract] | 3,109 |
| #17 | #12 or #13 or #14 or #15 or #16 | 82,567 |
| #18 | #11 and #17 | 27,016 |
| #19 | "Extracorporeal Membrane Oxygenation"[Mesh] | 16,406 |
| #20 | "Extracorporeal Membrane Oxygenation*"[Title/Abstract] | 18,948 |
| #21 | "ECMO"[Title/Abstract] | 14,355 |
| #22 | "extracorporeal cardiopulmonary resuscitation*"[Title/Abstract] | 1,199 |
| #23 | "ECPR"[Title/Abstract] | 1,010 |
| #24 | "extracorporeal life support*"[Title/Abstract] | 3,305 |
| #25 | "ECLS"[Title/Abstract] | 1,960 |
| #26 | #19 or #20 or #21 OR #22 or #23 or #24 or #25 | 27,008 |
| #27 | #18 and #26 | 2,037 |
| An updated search using the same strategies was conducted on March 20, 2025, which identified additional 165 records. | | |
| ***Web of Science*** | | |
|  | Query | Records on July 29^th^, 2024 |
| #1 | TS=("cardiac surg*") | 133962 |
| #2 | TS=("heart surg*") | 33511 |
| #3 | TS=("cardiac operati*") | 5982 |
| #4 | TS=("heart operati*") | 1937 |
| #5 | TS=("cardiopulmonary resuscitation*") | 51186 |
| #6 | TS=("CPR") | 33937 |
| #7 | TS=("Cardiopulmonary Bypass*") | 64905 |
| #8 | (#7 OR #6 OR #5 OR #4 OR #3 OR #2 OR #1) | 257689 |
| #9 | TS=("heart arrest*") | 51086 |
| #10 | TS=("cardiac arrest*") | 86184 |
| #11 | TS=("asystole*") | 6837 |
| #12 | TS=("cardiopulmonary arrest*") | 4995 |
| #13 | (#12 OR #11 OR #10 OR #9) | 109403 |
| #14 | (#8 AND #13) | 39338 |
| #15 | TS=("extracorporeal membrane oxygenation*") | 34348 |
| #16 | TS=("ECMO") | 23278 |
| #17 | TS=("extracorporeal cardiopulmonary resuscitation*") | 1725 |
| #18 | TS=("ECPR") | 1537 |
| #19 | TS=("extracorporeal life support*") | 4854 |
| #20 | TS=("ECLS") | 3495 |
| #21 | (#20 OR #19 OR #18 OR #17 OR #16 OR #15) | 42678 |
| #22 | (#21 AND #14) | 3224 |
| An updated search using the same strategies was conducted on March 20, 2025, which identified additional 258 records. | | |
| ***Cochrane Library*** | | |
|  | Query | Records on July 29^th^, 2024 |
| #1 | MeSH descriptor: [Cardiac Surgical Procedures] explode all trees | 17331 |
| #2 | (cardiac surg*):ti,ab,kw | 25260 |
| #3 | (heart surg*):ti,ab,kw | 40939 |
| #4 | (cardiac operati*):ti,ab,kw | 7831 |
| #5 | (heart operati*):ti,ab,kw | 14659 |
| #6 | MeSH descriptor: [Cardiopulmonary Resuscitation] explode all trees | 1724 |
| #7 | (Cardiopulmonary Resuscitation*):ti,ab,kw | 3138 |
| #8 | (CPR):ti,ab,kw | 2914 |
| #9 | MeSH descriptor: [Cardiopulmonary Bypass] explode all trees | 3180 |
| #10 | (Cardiopulmonary Bypass*):ti,ab,kw | 7875 |
| #11 | #1 or #2 or #3 or #4 or #5 or #6 or #7 or #8 or #9 or #10 | 67887 |
| #12 | MeSH descriptor: [Heart Arrest] explode all trees | 3018 |
| #13 | (Heart Arrest*):ti,ab,kw | 5351 |
| #14 | (cardiac arrest*):ti,ab,kw | 5761 |
| #15 | (asystole*):ti,ab,kw | 298 |
| #16 | (Cardiopulmonary Arrest*):ti,ab,kw | 2644 |
| #17 | #12 or #13 or #14 or #15 or #16 | 8140 |
| #18 | #11 and #17 | 4158 |
| #19 | MeSH descriptor: [Extracorporeal Membrane Oxygenation] explode all trees | 358 |
| #20 | (Extracorporeal Membrane Oxygenation*):ti,ab,kw | 972 |
| #21 | (ECMO):ti,ab,kw | 996 |
| #22 | (extracorporeal cardiopulmonary resuscitation*):ti,ab,kw | 104 |
| #23 | (ECPR):ti,ab,kw | 67 |
| #24 | (extracorporeal life support*):ti,ab,kw | 216 |
| #25 | (ECLS):ti,ab,kw | 67 |
| #26 | #19 or #20 or #21 OR #22 or #23 or #24 or #25 | 1559 |
| #27 | #18 and #26 | 125 |
| An updated search using the same strategies was conducted on March 20, 2025, which identified additional 17 records. | | |
| ***Ichushi Web*** | | |
|  | Query | Records on July 29^th^, 2024 |
| #1 | 心臓血管外科/TH | 25734 |
| #2 | 心臓血管外科/TA | 422 |
| #3 | 心臓外科/TA | 176 |
| #4 | 胸部外科学/TH | 1 |
| #5 | 胸部外科/TA | 110 |
| #6 | 心肺手術/TA | 1 |
| #7 | 心臓手術/TA | 535 |
| #8 | 心肺蘇生法/TH | 913 |
| #9 | 心肺蘇生/TA | 1373 |
| #10 | CPR/TA | 768 |
| #11 | 心肺バイパス術/TH | 1389 |
| #12 | 心肺バイパス/TA | 469 |
| #13 | 心臓カテーテル法/TH | 2341 |
| #14 | 心臓カテーテル/TA | 2018 |
| #15 | #1 or #2 or #3 or #4 or #5 or #6 or #7 or #8 or #9 or #10 or #11 or #12 or #13 or #14 | 30588 |
| #16 | 心停止/TH | 2699 |
| #17 | 心停止/TA | 2437 |
| #18 | 心臓停止/TA | 9 |
| #19 | 心拍停止/TA | 37 |
| #20 | 心肺停止/TA | 2138 |
| #21 | 心静止/TA | 153 |
| #22 | 心肺機能停止/TA | 49 |
| #23 | 心房停止/TA | 47 |
| #24 | 心肺蘇生法/TH | 913 |
| #25 | 心肺蘇生/TA | 1373 |
| #26 | #16 or #17 or #18 or #19 or #20 or #21 or #22 or #23 or #24 or #25 | 5552 |
| #27 | ECMO/TH | 942 |
| #28 | ECMO/TA | 854 |
| #29 | ECPR/TA | 24 |
| #30 | ECLS/TA | 13 |
| #31 | 人工肺/TH | 250 |
| #32 | 人工肺/TA | 649 |
| #33 | 人工心肺装置/TH | 1283 |
| #34 | 人工心肺装置/TA | 61 |
| #35 | 生命維持装置/TH | 3 |
| #36 | 生命維持装置/TA | 13 |
| #37 | 心臓補助機器/TH | 452 |
| #38 | 心臓補助/TA | 6 |
| #39 | 体外式心肺補助/TA | 9 |
| #40 | 体外式肺補助/TA | 25 |
| #41 | 体外心肺蘇生/TA | 1 |
| #42 | 体外循環式心肺蘇生/TA | 26 |
| #43 | #27 or #28 or #29 or #30 or #31 or #32 or #33 or #34 or #35 or #36 or #37 or #38 or #39 or #40 or #41 or #42 | 2977 |
| #44 | #15 and #26 and #43 | 460 |
| An updated search using the same strategies was conducted on March 20, 2025, which identified additional 18 records. | | |

Supplement C: Extracted data from included studies

| ***Extracted d*ata *from included studies*** |
| --- |
| Publication year and type, patient description, surgical procedures, intraoperative data, overall survival, survival with favorable neurological outcome, incidence of neurological and any other complications, duration of chest compression and ECMO after ECPR, and cannulation cite. Overall survival and survival with favorable neurological outcome, duration of chest compression, and distribution of cannulation site were plotted and visualized in graphs. |

**ECMO:** extracorporeal membrane oxygenation**; ECPR:** extracorporeal cardiopulmonary resuscitation.

Supplemental table S1: Adult studies with identifiable post-cardiac surgery ECPR subgroup (n < 20)

| Author | Year | Country | Publication type | Total cohort size* | Population | Age, years | Isolated CABG | Valve surgery | VAD surgery | MICS | **Post-cardiac surgery ECPR** | **Survival of post-cardiac surgery ECPR patients** | Neurological complication during ECMO in the study | Survival with favorable neurological outcome in the study | Detail of the favorable outcome | Other ECMO-related complications | Duration of chest compression, min | Cannulation site |
| --- | --- | --- | --- | --- | --- | --- | --- | --- | --- | --- | --- | --- | --- | --- | --- | --- | --- | --- |
| Bari | 2024 | Netherlands | Retrospective, observational | 39 | Post-cardiac surgery ECMO | 66 (52-72) | 15 (39) | 25 (64) | 0 (0) | 0 (0) | **17** | **2/17 (12)** | 11/39 (28) | 6/39 (15) | NA; 6 survivors did not have neurologic complication. | Re-exploration for bleeding 20 (51);c annulation site bleeding 5 (13); sepsis 10 (27); arrhythmia 19 (49); distal ischemia 5 (13); right ventricular failure 6 (15); AKI 23 (59); ARDS 2 (5) | NA | Central, 9 (23) peripheral 25 (64) Missing 5 (13) |
| Chen | 2003 | Taiwan | Retrospective, observational | 57 | ECPR including non-surgical patients | 57 ± 15 | NA | NA | NA | NA | **14** | **8/14 (57)** | NA | 17/57 (30) | NA; among all survivors (n=18) in the study, one had a "severe neurologic deficits." | MOF; cannulation-related bleeding; retroperitoneal hematoma in 1 (failed cannulation); limb ischemia leading to amputation in 1 survivor. | 48 ± 13 | NA |

Values are n, n (%), median (25%tile-75%tile), or mean (SD). Values are presented as reported in the primary studies and were not transformed. NA (not applicable) indicates the variable was not reported at the study/stratum level.

AKI: acute kidney injury; ARDS: acute respiratory distress syndrome; CABG: coronary artery bypass graft; ECMO: extracorporeal membrane oxygenation; ECPR: extracorporeal cardiopulmonary resuscitation; MICS: minimally invasive cardiac surgery; MOF: multiple organ failure; NA: not applicable; VAD; ventricular assist device.

*Include the total number of patients in the source study including non-ECPR patients.

Bolded values indicate estimates specific to the post–cardiac surgery (postcardiotomy) ECPR subgroup (i.e., calculated using the post–cardiac surgery ECPR denominator when extractable). All other values reflect the overall study cohort unless otherwise specified.

Supplemental table S2: Adult post–cardiac surgery ECPR case reports

| Author | Year | Country | Total # of patients in the report | Patients’ description | Age, years | Post-cardiac surgery ECPR | Cause of cardiac arrest | Survival of post-cardiac surgery ECPR patients | Neurological complication | Other ECMO-related complications | Duration of chest compression,　min | Cannulation site |
| --- | --- | --- | --- | --- | --- | --- | --- | --- | --- | --- | --- | --- |
| Doita | 2022 | Japan | 1 | Cardiac arrest 1-year after LVAD implantation due to thrombotic inflow obstruction | 48 | 1 | Thrombotic LVAD-inflow obstruction | 0 (0) | 1 (100) | Hypoxic encephalopathy  MOF | NA | Peripheral 1 (100) |
| Kitagawa | 2021 | Japan | 1 | Cardiac arrest due to tamponade with mediastinitis after AVR | 54 | 1 | Tamponade with mediastinitis | 1 (100) | NA | NA | NA | Peripheral 1 (100) |

Values are n, n (%).

AVR: aortic valve replacement; ECMO: extracorporeal membrane oxygenation; ECPR: extracorporeal cardiopulmonary resuscitation; LVAD; left ventricular assist device; MOF: multiple organ failure; NA: not applicable.

Supplemental table S3: Pediatric studies with identifiable post-cardiac surgery ECPR subgroup (n < 20)

| Authors | Year | Total cohort size* | Population | Age | Body  weight, kg | Single ventricular physiology | HLHS, or Norwood-type operation | **Post-cardiac surgery ECPR** | **Survival of post-cardiac surgery ECPR patients** | Survival with favorable neurological outcome in all patients in the study | **Survival with favorable neurological outcome in post-cardiac surgery ECPR** | Definition of the favorable neurological outcome | Other ECMO-related complications | Duration of chest compression, min | Cannulation site |
| --- | --- | --- | --- | --- | --- | --- | --- | --- | --- | --- | --- | --- | --- | --- | --- |
| Aharon | 2001 | 50 | Post-cardiac surgery mechanical support | 40 d (NA) | NA | 18 (36) | 14 (28) | **10** | **8/10 (80)** | 8/50 (16) | **8/10 (80)** | Discharged home | Re-exploration for bleeding 50 (100); hemodialysis 4 (8); pulmonary infection 6 (12); sepsis 7 (14); mediastinitis 4 (8) | Mean 45  (range 5-110) | Central 49 (98) |
| Balasubramanian | 2007 | 53 | Post-cardiac surgery ECMO | 150 (range 1–3960) d | 5.4  (range 2.4–30) | 3 (5) | NA | **10** | **5/10 (50)** | NA | **NA** | NA | Bleeding 36%; neurological 19%; sepsis 17%; renal 57%; pulmonary 21%; mechanical 47% | NA | Central 21%  Neck 79% |
| Dhillon | 2019 | 46 | Post-cardiac surgery IHCA | 2.9 (1.0-8.0) mo | 3.4 (2.6-6.5) | 19 (41) | 9 (20) | **10** | **5/10 (50)** | NA | **NA** | NA | NA | NA | NA |
| del Nido | 1992 | 11 | Post-cardiac surgery ECPR | 6 (1.2-13) mo | 5 (3.5-6.8) | NA | 0 (0) | **11** | **6/11 (55)** | NA | **NA** | NA | Bleeding 1 (1) Sepsis 1 (1) | 60 (42-90) | Peripheral 11 (100) |
| Deng | 2024 | 510 | Post-cardiac surgery ECMO | 301 ± 403 d | <20 kg  (inclusion criterion) | 105 (20) | 38 (7.5) | **8** | **5/8 (62)** | NA | **NA** | NA | NA | NA | Within 1-week post-cardiotomy: central; neck was used for peripheral. |
| Dohain | 2019 | 30 | Post-cardiac surgery ECMO | 6.5 (1–20) mo | 5 (3–9) | 12 (40) | 5 (17) | **10** | **2/10 (20)** | NA | **NA** | NA | Renal insufficiency 14 (46); liver dysfunction 10 (33); limb ischemia 3 (10); hemofiltration 20 (66); hemodialysis 5 (16) | 30 (14–93) | Central 29 (96)  Femoral 1 (3) |
| Guo | 2019 | 11 | Post-cardiac surgery ECPR | 1.5 (NA) mo | 3.9 (NA) | NA | 0 (0) | **11** | **4/11 (36)** | 4 (36) | **4 (36)** | Described as "All the post-op ECPR survivor showed favorable neurological outcome," and detail not applicable. | Renal failure 6 (55) Sepsis 2 (18) Circuit thrombus 2 (18) | 60 (NA) | Central 11 (100) |
| Gupta | 2015 | 52 | Post-cardiac surgery ECMO (≥7 days after surgery) | 64 (22-227) d | 3.5 (2.8, 6) | NA | 14(25) | **14** | **6/14 (42)** | NA | **NA** | NA | Dialysis 24 (44); positive blood culture 8/55 (15); hepatic insufficiency 3 (5); necrotizing enterocolitis 1 (2); bleeding complications 9 (16) | 41 (40-55) | Central 39 (71)  Neck 16 (29) |
| Hoskote | 2006 | 25 | Post-cardiac surgery ECMO | 15 (NA) d | 3.4 (NA) | 25 (100) | 18 (72) | **14** | **5 (36)** | NA | **NA** | NA | Re-exploration for bleeding 11 (44) Dialysis 11 (44) Sepsis 10 (25) Pulmonary complication 3 (12) Arrhythmias 7 (28) MOF 9 (36) | 36 (NA) | Central 24 (96)  Neck 1 (4) |
| Polimenakos | 2011 | 14 | Post-cardiac surgery ECPR (neonates) | 7.8 ± 2.9 d | 3.4 ± 1.8 | 14 (100) | 10 (71) | **14** | **8/14 (57)** | NA | **NA** | NA | Bleeding 3 (21) Dialysis 2 (14) Sepsis or necrotizing enterocolitis 5 (36) MOF 4 (29) | 39 (26-52) | NA |
| Sarıoğlu | 2014 | 10 | Post-cardiac surgery mechanical support | 5 (3-12) mo | NA | 2 (20) | 0 (0) | **2** | **2/2 (100)** | 4/10 (40) | **2/2 (100)** | All the four survivors discharged without permanent injury; normal growth on follow-up. | NA | NA | NA |
| Tsukahara | 2014 | 21 | ECPR including non-surgical patients | 0 (0-33) mo | 4 (3-12) | 9 (43) | 1 (5) | **11** | **2/11 (18)** | 2/21 (10) | **2/11 (18)** | Described as "favorable neurologic outcome," detail not applicable. | NA | 60 (NA) | Central 15 (71) |
| Kramer | 2020 | 72 | ECPR including non-surgical patients | 0.3 (0.04–1.9) y | 4.5 (3–10) | 32 (44) | NA | **42** | **19/42 (45)** | 19/72 (26) | **NA** | ΔPCPC ≤ 1 vs prearrest | Bleeding 34 (47) (re-exploration 16); oxygenator change 14 (19); clot formation requiring circuit/cannula revision 9 (12); infections 6 (8); AKI 22 (30); renal replacement therapy 22 (30); hepatic dysfunction 26 (36) | 60 (42–80) | Central 59 (82)  Neck 8 (11)  Femoral 5 (7) |
| Ozturk | 2023 | 26 | ECPR including non-surgical patients | 60 (26–141) mo | NA | NA | NA | **11** | **4/11 (36)** | 9/26 (35) | **NA** | PCPC ≤2 | Bleeding 20 (77); renal failure 17 (65); hepatic failure 5 (19); limb ischemia 2 (8); bloodstream infections 4 (15); CRRT 13 (50) | Survived to discharge 69 ± 5; did not survive 72 ± 4 | Central 24 (92)  Peripheral (femoral) 2 (8) |
| Huang | 2008 | 27 | ECPR including non-surgical patients | 4.4 y (range 6 d–17 y) | NA | NA | NA | **11** | **3/11 (27)** | 10/27 (37) | **NA** | PCPC 1–3 or no change from admission | Renal failure after ECPR 12 (44) | All cohort 50 (30–60); survivors 45 (range 10–90); nonsurvivors 60 (range 22–250) | Central  (post-cardiac surgery) 11 (41) Peripheral 16 (59)  (Neck 12; femoral 4) |
| Hamrick | 2003 | 53 | Post-cardiac surgery ECMO | Survivors 27 (range 1–362) d; nonsurvivors 22 (range 1–314) d | Survivors 4.3 (3.5–6)  Nonsurvivors 3.9 (2.1–7.9) | NA | 4 (7) | **12** | **1/12 (8)** | 7/53 (13) | **NA** | Cognitive abnormal if >2 SD below; neuromotor abnormal if functional limitation/cerebral palsy. The seven survivors were completely intact. | Continuous arteriovenous hemofiltration34 (64) | NA | NA |

Values are n, n (%), median (25%tile-75%tile or range), or mean (SD). Values are presented as reported in the primary studies and were not transformed. All studies listed were retrospective/observational. NA (not applicable) indicates the variable was not reported at the study/stratum level.

AKI: acute kidney injury; CRRT: continuous renal replacement therapy; d: days; ECMO: extracorporeal membrane oxygenation; ECPR: extracorporeal cardiopulmonary resuscitation; mo: months, MOF: multiple organ failure; NA: not applicable; PCPC: pediatric cerebral performance category scale; SD: standard deviation; y: years.

*Include the total number of patients in the source study including non-ECPR patients.

Bolded values indicate estimates specific to the post–cardiac surgery (postcardiotomy) ECPR subgroup (i.e., calculated using the post–cardiac surgery ECPR denominator when extractable). All other values reflect the overall study cohort unless otherwise specified.

Supplemental table S4: : Pediatric post–cardiac surgery ECPR case reports/series (including small cohort study [ECPR n<10])

| Authors | Year | Country | Total cohort size* | Patients’ description | Age | Body  weight, kg | Single ventricular physiology | HLHS, or Norwood-type operation | **Post-cardiac surgery ECPR** | **Survival of post-cardiac surgery ECPR patients** | Survival with favorable neurological outcome in all patients in the study | **Survival with favorable neurological outcome in post-cardiac surgery ECPR** | Detail of neurological outcome | Other ECMO-related complications | Duration of chest compression, min | Cannulation site |
| --- | --- | --- | --- | --- | --- | --- | --- | --- | --- | --- | --- | --- | --- | --- | --- | --- |
| Alghamdi | 2010 | Canada | 1 | Cardiac arrest due to massive air embolism after TAPVD repair | 0 d | 3.5 | NA | 0 | **1** | **1 (100)** | 1 (100) | **1 (100)** | No clinical or radiological evidence of neurologic damage. | No complication | NA | Central 1 (100) |
| Amberman | 2010 | USA | 1 | Cardiac arrest after Norwood procedure | 1 mo | 3.2 | 1 (100) | 1 (100) | **1** | **1 (100)** | 0 (0) | **0 (0)** | A few seizure-like episode, MRI showed global hypoxic hypoperfusion injury, but extubated and later underwent Glenn, and discharged home. No further detail about neuro findings. | Global hypoxic hypoperfusion brain injury on MRI but "clinically doing well" | NA | Peripheral 1 (100) |
| Bakos | 2021 | Croatia | 1 | Cardiac arrest after Blalock-Taussig shunt procedure | 2 w | 3.8 | 1 (100) | 0 (0) | **1** | **1 (100)** | 1 (100) | **1 (100)** | Described as “”no complication.” | No complication | 120 | Central 1 (100) |
| Gutiérrez-Soriano | 2023 | Colombia | 1 | Cardiac arrest after ASD and VSD closure | 4 mo | NA | 0 (0) | 0 (0) | **1** | **1 (100)** | 1 (100) | **1 (100)** | Mild abnormally low muscle tone was observed at discharge, without other neurological deficits, and with proper extremity movement. | Mild low muscle tone without other neurological deficits, and with proper extremity movement | 60 | Central 1 (100) |
| Hasegawa | 2016 | Japan | 1 | Cardiac arrest after TAPVD repair | 39 w | NA | 1 (100) | 0 (0) | **1** | **0 (0)** | NA | **NA** | NA | Died due to pulmonary CMV infection | NA | NA |
| Jaggers | 2000 | USA | 35 | Children after congenital heart surgery requiring postcardiotomy ECMO | 19 (range 1–820) d | Survivors 4.8 kg; nonsurvivors 4.5 | 10 (35) | 5 (14) | **6** | **3/6 (50)** | NA | **3/6 (50)** | All ECPR survivors reported as "neurologically normal." | Coagulopathy 15 (42); infection 11 (31); renal failure 9 (25); pulmonary 12 (34); thrombotic 7 (20) | NA | NA |
| Kendirli | 2021 | Turkey | 15 | Pediatric (<age 18 y) ECPR | 60 (4–156) mo | 18 (4.8–145) | NA | NA | **7** | **4/7 (57)** | 5/15 (33) | **4/7 (57)** | PCPC ≤ 2 | CRRT 5; DIC-related bleeding deaths 4 | 95 (20–320)  (low-flow time) | Central 2 (13); peripheral 10 (67) |
| Pizarro | 2001 | USA | 12 | Neonates who underwent stage I Norwood palliation and received ECMO | 3.9 (range 1–14) d | 2.6 (1.4–3.8) | 12 (100) | 12 (100) | **2** | **0/2 (0)** | 6 (50) | **0/2 (0)** | NA | Sepsis 5 (41); renal failure 5 (41); neurologic 3 (25); respiratory 3 (25); bleeding 2 (16); circuit complication 1 (8) | NA | Central 12 (100) |
| Zobel | 1994 | Austria | 1 | Cardiac arrest after VSD closure and RVOT reconstruction | 2 y | NA | 1 (100) | 0 (0) | **1** | **1 (100)** | 1 (100) | **1 (100)** | Described as “patient showed good neurologic recovery and could be discharged.” | CRRT, DIC | NA | Central 1 (100) |

Values are n, n (%), median (25%tile-75%tile or range), or mean (SD). Values are presented as reported in the primary studies and were not transformed. All studies listed were retrospective/observational. NA (not applicable) indicates the variable was not reported at the study/stratum level.

ASD: atrial septal defect; te kidney injury; CMV: cytomegalovirus; CRRT: continuous renal replacement therapy; d: days; DIC: disseminated intravascular coagulation; ECMO: extracorporeal membrane oxygenation; ECPR: extracorporeal cardiopulmonary resuscitation; TAPVD: total anomalous pulmonary venous drainage; mo: months, MOF: multiple organ failure; MRI: magnetic resonance imaging; NA: not applicable; PCPC: pediatric cerebral performance category scale; SD: standard deviation; VSD: ventricular septal defect; y: years.

*Include the total number of patients in the source study including non-ECPR patients.

Bolded values indicate estimates specific to the post–cardiac surgery (postcardiotomy) ECPR subgroup (i.e., calculated using the post–cardiac surgery ECPR denominator when extractable). All other values reflect the overall study cohort unless otherwise specified.

Supplemental table S5: Definition of favorable neurological outcome and reported complications during ECMO in pediatric studies with identifiable post-cardiac

surgery ECPR subgroup (n ≥ 20)

| Authors/Year | Definition of the favorable neurological outcome | ECMO-related complications* |
| --- | --- | --- |
| Alsoufi/2014 | NA | Re-exploration for bleeding 17 (44); Dialysis 19 (49); Sepsis 7 (18); Pulmonary hemorrhage 6 (15); GI bleeding 3 (8); Mechanical problems 2 (6) |
| Basgoze /2022 | NA | Surgical or cannulation site bleeding 24 (35); DIC 11 (16); Sepsis 34 (51); Peritoneal dialysis 87 (80); Hemodialysis 74 (68); VAC therapy 13 (12); Tracheostomy 13(12) |
| Chan/2008 | NA | Cannula bleeding 151 (31); Dialysis 43 (8.7); Sepsis 42 (9); Pulmonary hemorrhage 37 (8); GI bleeding 7 (1.4); Hyperbilirubinemia 25 (5); Arrhythmias 92 (18.7); CPR 29 (6) |
| Kobayashi/2024 | NA | NA |
| Melvan/2020 | Definition not applicable. All survivors: 79 patients, of which 74 had no neuro-complication. Post-cardiac surgery ECPR survivor: 53 patients, of which 49 had no neuro-complication. | Re-exploration for bleeding 63 (34); Dialysis 74 (40); Mechanical complications 41 (2); Pulmonary hemorrhage 8 (4) |
| Shah/2005 | NA | Cannulation/surgical site bleeding 36 (43); Oxygenator replacement 3 (4); Circuit thrombus 16 (19) |
| Walter/2011 | NA | Re-exploration for bleeding 5 (12); Sepsis 1 (2); Mediastinitis 1 (2);  Massive consumption coagulopathy 5 (12) |
| Wolf/2012 | NA | NA |
| Brown/2023 | PCPC ≤2 or unchanged from baseline | NA |
| Jin/2020 | NA | Hemorrhage 59 (69); Thrombosis 26 (30); Hemolysis 42 (49); Nosocomial infection 34 (40); AKI 43 (50); Hyperbilirubinemia 40 (47); Thrombocytopenia 27 (31); Hypoalbuminemia 31 (36); Anemia 21 (24); ECMO system replacement 11 (12) |
| Torres-Andres/2018 | NA | Circuit thrombosis requiring circuit change 1 |
| Beshish/2018 | Δfunctional status scale (admission vs discharge) <5 | Chest re-exploration 43 (54); inhaled nitric oxide 15 (19); high-frequency oscillatory ventilation 4 (5); ventricular assist device 1 (1) |
| Erek/2017 | NA | Renal dysfunction 22 (88); bleeding 5 (20); ventricular dysfunction 5 (20); sepsis/multiorgan failure 4 (16); low cardiac output 3 (12); mediastinitis 1 (4); purpura fulminans 1 (4) |
| Huang/2012 | PCPC 1–3 or no change from admission | Renal failure: 19 (66) in non-survivors, 5 (20) in survivors |
| Kane/2010 | PCPC ≤2 | ECMO circuit complications 59 (34); Surgical/cannulation site bleeding 65 (37); Respiratory complications 31 (18); Sepsis 38 (22); Renal failure 53 (30); Dialysis 18 (10); Liver injury 20 (11) |
| Alsoufi/2009 | NA | Re-exploration for bleeding 100 (56); Renal dysfunction 18 (10); Pulmonary hemorrhage 9 (5); Mechanical problems requiring circuit component changes 68 (37) |
| Anton-Martin/2020 | PCPC ≤2 at discharge | Continuous renal replacement therapy 27 (37) |

Values are n, n (%).

AKI: acute kidney injury; CPR: cardiopulmonary resuscitation; DIC: disseminated intravascular coagulation; ECMO: extracorporeal membrane oxygenation; ECPR: extracorporeal cardiopulmonary resuscitation; GI: gastrointestinal; NA: not applicable; PCPC: pediatric cerebral performance category scale; VAC: vacuum-assisted closure.

*Including non-ECPR patients.
